# GSK-3 inhibitors induce chromosome instability

**DOI:** 10.1186/1471-2121-8-34

**Published:** 2007-08-14

**Authors:** Anthony Tighe, Arpita Ray-Sinha, Oliver D Staples, Stephen S Taylor

**Affiliations:** 1 Faculty of Life Sciences, Michael Smith Building, Oxford Road, University of Manchester, Manchester M13 9PT, UK; 2 Division of Surgery and Oncology, University of Liverpool, 5^th ^Floor UCD Building, Daulby Street, Liverpool, L69 3GA, UK; 3 Department of Surgery and Molecular Oncology, Ninewells Hospital, University of Dundee, Dundee DD1 9SY, UK

## Abstract

**Background:**

Several mechanisms operate during mitosis to ensure accurate chromosome segregation. However, during tumour evolution these mechanisms go awry resulting in chromosome instability. While several lines of evidence suggest that mutations in *adenomatous polyposis coli *(*APC*) may promote chromosome instability, at least in colon cancer, the underlying mechanisms remain unclear. Here, we turn our attention to GSK-3 – a protein kinase, which in concert with APC, targets β-catenin for proteolysis – and ask whether GSK-3 is required for accurate chromosome segregation.

**Results:**

To probe the role of GSK-3 in mitosis, we inhibited GSK-3 kinase activity in cells using a panel of small molecule inhibitors, including SB-415286, AR-A014418, 1-Azakenpaullone and CHIR99021. Analysis of synchronised HeLa cells shows that GSK-3 inhibitors do not prevent G1/S progression or cell division. They do, however, significantly delay mitotic exit, largely because inhibitor-treated cells have difficulty aligning all their chromosomes. Although bipolar spindles form and the majority of chromosomes biorient, one or more chromosomes often remain mono-oriented near the spindle poles. Despite a prolonged mitotic delay, anaphase frequently initiates without the last chromosome aligning, resulting in chromosome non-disjunction. To rule out the possibility of "off-target" effects, we also used RNA interference to selectively repress GSK-3β. Cells deficient for GSK-3β exhibit a similar chromosome alignment defect, with chromosomes clustered near the spindle poles. GSK-3β repression also results in cells accumulating micronuclei, a hallmark of chromosome missegregation.

**Conclusion:**

Thus, not only do our observations indicate a role for GSK-3 in accurate chromosome segregation, but they also raise the possibility that, if used as therapeutic agents, GSK-3 inhibitors may induce unwanted side effects by inducing chromosome instability.

## Background

Genome stability requires that the replicated chromosomes are accurately segregated during mitosis [1]. Chromosome segregation is mediated by a microtubule spindle, to which chromosomes attach via their kinetochores, complex microtubule-binding structures which assemble at the centromeric heterochromatin [2-4]. Kinetochores not only attach chromosomes to the spindle, they also perform two key functions which maintain chromosome stability. Firstly, by undergoing rounds of microtubule capture-and-release, kinetochores select microtubule attachments which yield tension across the centromere [5]. This in turn promotes chromosome biorientation, i.e. sister kinetochores attached to opposite spindle poles. Secondly, by monitoring microtubule occupancy and/or tension, kinetochores regulate the spindle checkpoint, a surveillance mechanism which delays anaphase until all the chromosomes are bioriented [6].

As a consequence of these mechanisms, most normal proliferating human cells are diploid and karyotypically stable. By contrast, many tumour cells exhibit chromosome instability and are therefore karyotypically unstable and aneuploid [7]. Much effort has gone into defining the genetic lesions responsible for the chromosome instability and recently, *adenomatous polyposis coli *(*APC*) has emerged as a candidate, at least in colon cancer [8,9]. APC is best known for its role in the Wnt signalling pathway: in the absence of Wnt signals, a destruction complex of APC and axin recruits both β-catenin and GSK-3 [10,11]. Phosphorylation of β-catenin by GSK-3β then targets β-catenin for proteolysis. In the presence of Wnt signals, β-catenin phosphorylation is inhibited, resulting in the upregulation of proliferative genes. This mechanism is essential for tumour suppressor function in the colonic epithelia: almost all colon cancers have either loss of function mutations in APC or activating mutations in β-catenin [12]. However, APC is a large multi-domain protein and its function is not restricted to the Wnt pathway.

Evidence is mounting that APC is somehow required for the fidelity of chromosome segregation. APC is a microtubule binding protein and has the ability to stabilise plus ends [13]. In mitosis, APC localises to kinetochores in a microtubule dependent manner [14,15], and tumour cells with APC mutations have weaker kinetochore – microtubule interactions [16,17]. Spindles assembled in *Xenopus *egg extracts depleted of APC are abnormal [18]. APC also localises to centrosomes [19-21], and in the *Drosophila *germ line, APC is required for spindle positioning [22]. In mice, APC mutation enhances genomic instability and tumour formation in cells haploinsufficient for BubR1, a spindle checkpoint kinase [23]. Murine embryonic stem cells with APC mutations are frequently tetraploid [14,15]. Ectopic expression of N-terminal APC mutants in diploid, APC-proficient human cells compromises the spindle checkpoint and enhances survival following prolonged mitotic arrest, leading to aneuploidy [21]. However, despite this body of evidence, the molecular mechanisms linking APC and chromosome instability remain unclear.

One possibility is that APC mutation compromises EB1, a microtubule tip-tracking protein involved in microtubule dynamics, spindle positioning, chromosome stability and cytokinesis [24,25]. EB1 binds the C-terminus of APC [26], so it is conceivable that the binding of N-terminal APC mutants to partners, including full length APC, excludes EB1 from complexes required for microtubule processes [17]. Another possible mechanism lies with, GSK-3. Like APC, the function of GSK-3 is not restricted to the Wnt pathway, rather it has been implicated in a plethora of processes including glycogen metabolism and tau phosphorylation [27-29], and more recently, regulating kinesin-driven organelle movement [30] and Cyclin E degradation [31]. Importantly, GSK-3 has been implicated in regulating interphase microtubule dynamics [32]. Phosphorylated GSK-3 localises to spindle poles in mitosis [33]. In both budding and fission yeast, overexpression of GSK-3 suppresses mutations in kinetochore proteins and the centromeric DNA [34,35]. Interestingly, Bub1 and BubR1, two related protein kinases which localise to kinetochores and are required for spindle checkpoint function [36], phosphorylate APC *in vitro *[15]. While the significance of this is unclear, phosphorylation is enhanced if APC is already phosphorylated by GSK-3 [15]. In one study, small molecule GSK-3 inhibitors were shown to induce spindle defects and chromosome misalignment [33]. However, whether these chromosomes eventually aligned before anaphase onset is unclear. Indeed, we envision two possible scenarios; either the chromosomes never align, yielding prolonged checkpoint arrest followed by adaptation and mitotic exit without dividing, yielding tetraploid cells [37]; or alternatively, the spindle checkpoint "gives up" and the cells enter anaphase despite unaligned chromosomes, thereby missegregating only those chromosomes [38].

To distinguish between these possibilities and further probe the role of GSK-3 in mitotic chromosome segregation, we characterised a panel of structurally diverse GSK-3 inhibitors. We show that these inhibitors have profound effects on cell cycle control, spindle morphology and chromosome alignment. Strikingly, these inhibitors increase the frequency with which cells missegregate their chromosomes. In support of the notion that these phenotypes are due to inhibition of GSK-3, rather than off target effects, we use RNA interference to selectively repress GSK-3β. This results in a chromosome alignment defect and the formation of micronuclei, a hallmark of chromosome missegregation.

## Results

### Characterisation of small molecule GSK-3 inhibitors

**Figure 1:**
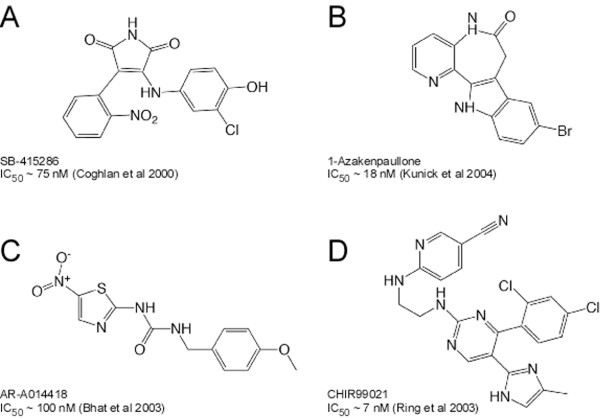
**Small molecule inhibitors of GSK-3**. Chemical structures of SB-415286, 1-Azakenpaullone, AR-A014418 and CHIR99021

**Figure 2:**
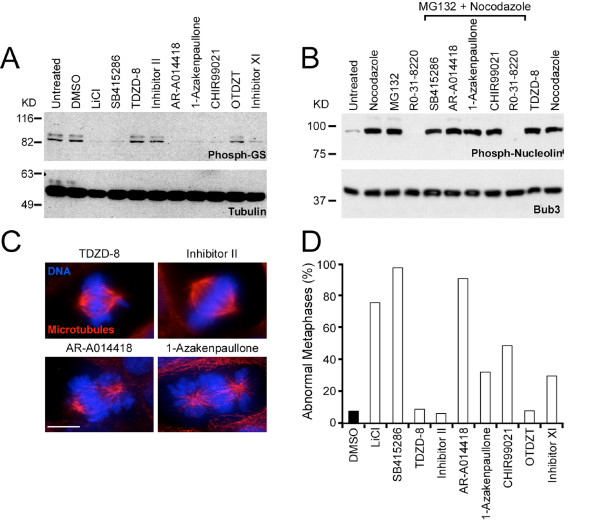
**Identification of small molecules which inhibit GSK-3 *in vivo***. HeLa and DLD-1 cells were incubated with small molecule GSK-3 inhibitors for 24 hours then analysed by immunoblotting and immunofluorescence. (**A**) Immunoblot of HeLa cell extracts showing inhibition of glycogen synthase phosphorylation by a subset of GSK-3 inhibitors. (B) Immunoblot of HeLa cell extracts showing that GSK-3 inhibitors do not inhibit Cdk1 phosphorylation of nucleolin. (**C**) Images of mitotic DLD-1 cells showing examples of abnormal metaphase spindles following exposure to AR-A014418 and 1-Azakenpaullone. (**D**) Bar graph plotting the number of abnormal metaphases in cells treated with different small molecule GSK-3 inhibitors.

A number of small molecules have been identified which inhibit GSK-3 *in vitro *[27]. To determine whether GSK-3 is required for accurate chromosome segregation, we obtained a panel of structurally diverse inhibitors (Fig. 1 and Table 1) and tested their ability to inhibit glycogen synthase (GS) phosphorylation in cells [39,40]. As a negative control, we used DMSO, the solvent in which the drugs were dissolved, and as a positive control we used lithium chloride, a well established GSK-3 inhibitor [41,42]. When HeLa cells were treated with GSK-3 inhibitors for 24 hours, only SB-415286, AR-A014418, 1-Azakenpaullone, CHIR99021 and Inhibitor XI significantly reduced GS phosphorylation (Fig. 2A). TDZD-8, Inhibitor II and OTDZT had little effect (Fig. 2A), even when used at relatively high concentrations (not shown).

**Table 1:** GSK-3 inhibitors used in this study

**Compound**	**Class**	**Mechanism**	**In vitro IC_50_**	**Conc. used in cells**	**Ref.**
SB-415286	Arylindole-maleimides	ATP Competitor	78 nM	30 μM	[44]
AR-A014418	Thiazole	ATP Competitor	104 nM	20 μM	[58]
1-Aza kenpaullone	Benzazepinone	ATP Competitor	18 nM	2.5 μM	[57]
CHIR99021	Aminopyrimidine	ATP Competitor	7 nM	10 μM	[47]
Lithium	Divalent ion	Non competitive	2 mM	40 mM	[41]
TDZD-8	Thiadiazolidinone	Non competitive	2 μM	10–60 μM	[70]
Inhibitor II	Pyridyloxadiazole	-	390 nM	25–75 μM	[71]
OTDZT	Thiadiazolidinone	Non competitive	10 μM	50–100 μM	[70]
Inhibitor XI	Azaindolylmaleimide	ATP Competitor	32 nM	50 μM	[72]

Several GSK-3 inhibitors have been shown to inhibit members of the CDK family, at least *in vitro *[27]. We therefore asked whether cellular Cdk1 activity was inhibited by the GSK-3 inhibitors used here. HeLa cells were synchronised in mitosis with nocodazole then exposed to GSK-3 inhibitors. In parallel, cells were exposed to a *bone fide *Cdk1 inhibitor, RO-31-8220 [43]. To prevent mitotic exit, the proteasome inhibitor MG132 was also added to the cells. Cell lysates were then prepared and immunoblotted for phospho-nucleolin, a known Cdk1 substrate (Fig. 2B). While RO-31-8220 clearly inhibited the phosphorylation of nucleolin, the GSK-3 inhibitors had no apparent effect. Thus, at the concentrations where GSK-3 is clearly inhibited (Fig. 2A), there does not appear to be any appreciable inhibition of Cdk1.

Next, we analysed spindle morphology in drug-treated mitotic DLD-1 cells. In controls, or cells treated with compounds which did not inhibit GS phosphorylation, normal metaphase spindles were readily apparent (Fig. 2C). By contrast, cells treated with compounds which did inhibit GS phosphorylation, frequently exhibited abnormal chromosome configurations. Specifically, although bipolar spindles formed and most chromosomes aligned at the metaphase plate, some chromosomes were often clustered near the spindle poles (Fig. 2C). For example, in the presence of the highly specific GSK-3 inhibitor, CHIR99021, ~49% of metaphases had one or more chromosomes clustered near the spindle poles (Fig. 2D). Thus, five structurally diverse small molecules which inhibit GSK-3 activity in cells induced a chromosome alignment defect.

### GSK-3 inhibitors delay mitotic entry and exit

**Figure 3:**
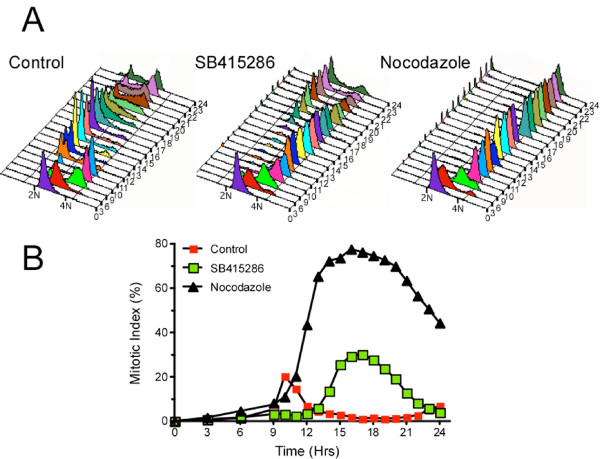
**GSK-3 inhibitors delay mitotic entry and exit**. HeLa cells were synchronised at G1/S then released into drug-free medium or medium containing either SB-415286 or nocodazole. At the time points indicated the cells, were harvested and analysed by flow cytometry to determine DNA content and mitotic index. (**A**) DNA content histograms showing that SB-415286 delays cell division. (**B**) Graph plotting the mitotic index, as determined by MPM-2 reactivity, showing that mitotic progression is delayed in SB-415286 treated cells.

Although GSK-3 inhibitors have been used extensively to study insulin action [44-47], very little is know about their effects on the cell cycle. In light of the observation described above, we therefore analysed the effect of GSK-3 inhibitors on cell cycle progression. HeLa cells were synchronised in early S-phase using a double thymidine block then released into drug free media, or media supplemented with SB-415286 or nocodazole. Over the next 24 hours cells were harvested at regular intervals and analysed by flow cytometry to determine DNA content and mitotic index. Control cells progressed through S-phase then entered mitosis 9 hours after release (Fig. 3A, B). The mitotic index peaked at ~20% 10 hours post-release then, as division was completed, cells with 2N DNA contents reappeared. 17 hours post-release, i.e. 5–6 hours after returning to G1, these cells entered a second S-phase. Cells released into nocodazole completed S-phase but then, consistent with hyper-activation of the spindle checkpoint, arrested in mitosis maintaining 4N DNA contents. Cells released into SB-415286 also progressed through the first S-phase will normal kinetics (Fig. 3A) but the mitotic index did not increase until 13 hours post-release, indicating a mitotic entry delay of ~3 hours (Fig. 3B). 17 hours post-release, the mitotic index peaked at ~30%. After this, the number of cells with 2N DNA contents started to increase, indicating successful cell division. By 23 hours, i.e. ~6 hours after they returned to G1, SB-415286-treated cells entered a second S phase. Thus in HeLa cells, SB-415286 has no apparent effect on G1/S progression and neither does it prevent cell division. SB-415286 does however delay mitotic entry and mitotic exit.

### GSK-3 inhibitors delay chromosome alignment

**Figure 4:**
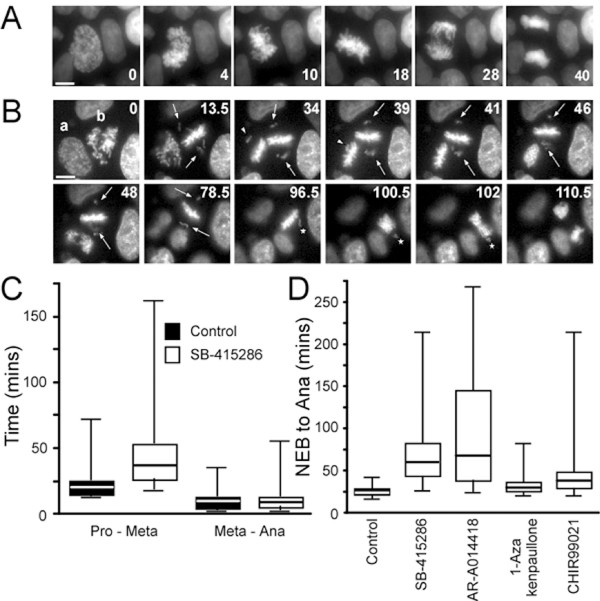
**GSK-3 inhibitors delay chromosome alignment**. DLD-1 cells expressing a tagged GFP-Histone H2B were incubated in the presence or absence of small molecule GSK-3 inhibitors then analysed by time-lapse microscopy. (**A**) Control cell showing normal mitosis. (**B**) SB-415286-treated cells. While cell ***a ***completes mitosis normally, chromosome alignment in cell ***b ***is delayed (see arrows) and anaphase is initiated despite one unaligned chromosome (see *). (**C**) Box plot measuring time from prophase to metaphase and metaphase to anaphase for at least 42 cells. (**D**) Box plot measuring time taken from nuclear envelope breakdown (NEB) to anaphase onset. Values taken from at least 41 cells.

To determine why SB-415286 delayed mitotic progression, we analysed drug-treated DLD-1 cells by time-lapse microscopy, using a GFP-tagged histone to visualise the chromosomes [38]. Control cells typically aligned their chromosomes within ~22 minutes (Table 2) then underwent a normal anaphase (Fig. 4A). Note that untreated cells never entered anaphase with unaligned chromosomes (Table 3). By contrast, chromosome alignment was often delayed in SB-415286 treated cells and anaphase was often initiated with unaligned chromosomes. For example, Figure 4B shows two cells, marked ***a ***and ***b***, which, at T = 34 minutes still have unaligned chromosomes (one in ***a***, and two in ***b***, see arrow head and arrows respectively). At T = 39 minutes, the last chromosome in cell ***a ***aligns and anaphase initiates at T = 46. By T = 96.5 minutes, one of the laggards in cell ***b ***has congressed, but one remains unaligned (see asterisk). At T = 100.5, cell ***b ***enters anaphase, without the last chromosome aligning.

**Table 2:** Effect of SB-415286 inhibitors on mitotic timing

**Compound**	**Event**	**Time (mins)**	**s.e.m.**	**Range**	**N**	**P**
Untreated	NEB-Metaphase	21.59	1.49	12–72	41	-
	Metaphase-Anaphase	10.65	0.92	1.5–35.5	49	-
SB-415286	NEB-Metaphase	46.04	4.32	17.5–162	42	p < 0.01
	Metaphase-Anaphase	11.32	1.2	1.5–55	57	p > 0.05

Values represent the mean time, in minutes, taken from nuclear envelope breakdown to metaphase and metaphase to anaphase in minutes. Also shown is showing the standard error of the mean, the range and the number of cells analysed. P values determined using a Kruskal-Wallis test.

**Table 3:** Effect of GSK-3 inhibitors on mitotic timing and chromosome non-disjunction

**Compound**	**Time (mins)**	**s.e.m.**	**Range**	**N**	**#**	**P**
-	25.37	0.35	16–42	189	0	-
SB4	69.56	3.44	26–214	99	15	p < 0.001
AR	92.88	10.02	24–268	41	3	p < 0.001
Ken	31.87	0.48	20–82	348	2	p < 0.001
CHIR	43.94	1.93	20–214	187	8	p < 0.001

Values represent the mean time, in minutes, taken from nuclear envelope breakdown to metaphase, Also shown is the standard error of the mean, the range, the number of cells analysed (N), the number of cells which enter anaphase with unaligned chromosomes (#). P values determined using a Kruskal-Wallis test.

To quantitate these effects, we measured the time taken from prophase to metaphase, i.e. when the last chromosome aligned, and the time taken from metaphase to anaphase. While control cells took 21.6 ± 1.4 minutes to reach metaphase, SB4-treated cells took, on average, 46.0 ± 4.3 minutes (Fig. 4C, Table 2). Anaphase initiated ~11 minutes later in both control (10.7 ± 0.9 minutes) and SB-415286-treated (11.3 ± 1.2 minutes) cells. Importantly however, six out of thirty-two SB-415286 treated cells entered anaphase with one or more unaligned chromosomes. This phenomenon was not restricted to SB-415286. In a separate set of experiments, we measured the time from nuclear envelope breakdown (NEB) to anaphase in the presence of SB-415286, AR-A014418, 1-Azakenpaullone and CHIR99021. Like SB-415286, AR-A014418 induced a prolonged mitotic delay (NEB to anaphase, 92.9 ± 4 minutes, Fig. 4D, Table 3). Following chromosome alignment, anaphase initiated with normal kinetics (not shown), confirming that the prolonged mitosis was due to delayed chromosome alignment. 1-Azakenpaullone and CHIR99021 induced modest but never-the-less significant (p < 0.001) delays, (Fig. 4D, Table 3). Importantly, a proportion of the cells exposed to AR-A014418, 1-Azakenpaullone and CHIR99021 entered anaphase with unaligned chromosomes (Table 3).

To see if such chromosome missegregation events would lead to aneuploidy, diploid HCT116 cells were exposed SB-415286 for 48 hours, metaphase spreads prepared then chromosome number determined. At day 0, the majority of untreated control cells had a near diploid karyotype, with only 3% of the cells having more than 49 chromosomes (not shown). By contrast, 13.4% of cells treated with SB-415286 had more than 49 chromosomes. By day 5, the aneuploid population (>49 chromosomes) in control cells was 5.6%, however in SB-415286 treated cells, 26.3% were now aneuploid (not shown).

Thus, when exposed to GSK-3 inhibitors, chromosome alignment is delayed and anaphase is often initiated in the presence of unaligned chromosomes yielding aneuploid cells.

### GSK-3 inhibitors induce mono-orientations

**Figure 5:**
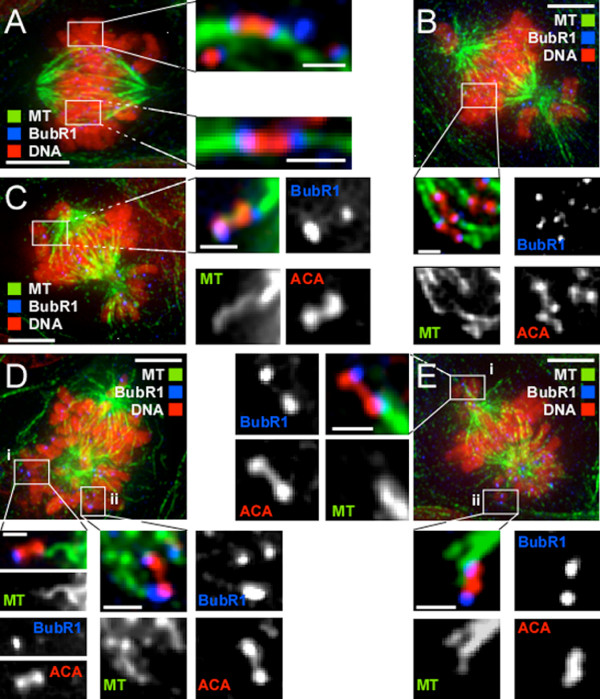
**GSK-3 inhibitors yield monopolar and syntelic attachments**. DLD-1 cells were incubated with GSK-3 inhibitors for 24 hours then fixed and stained to detect DNA (red), tubulin (green), BubR1 (blue) and centromeres (ACA, red) as indicated. Images represent projections of deconvolved image stacks. (**A**) Control cell showing examples of bioriented chromosomes. (**B**) GSK-3 inhibitor treated cell showing that chromosomes near the metaphase plate are bioriented. (**C-E**) Inhibitor treated cells showing examples of either syntelic attachments (C, Dii, Eii) or monopolar attachments (Di, Ei). Bar is 5 μm when the entire spindle is shown or 1 μm in the enlargements. The cells in C-E were treated with 30 μM SB-415286.

To determine why complete chromosome alignment was delayed in cells treated with GSK-3 inhibitors, we analysed K-fibres using high-resolution optical sectioning microscopy. To visualise centromeres and kinetochores, we stained cells with anti-centromere antibodies (ACA) and anti-BubR1 antibodies respectively. In control cells, chromosomes near the spindle equator were clearly bioriented: microtubules terminated at BubR1 foci which were connected by ACA staining (Fig. 5A). In drug-treated cells, chromosomes near the spindle equator also appeared to be correctly bioriented (Fig. 5B). However, chromosomes near the spindle poles were typically mono-oriented, with one kinetochore attached to the nearest spindle pole and the other unattached (Panels ***i ***in Fig. 5D, E). In some cases, microtubules from the same pole could be seen extending towards both kinetochores of an unaligned chromosome, indicating a possible syntelic orientation (Fig. 5C and panels ***ii ***in 5D, E). Thus, while GSK-3 inhibitors do not prevent kinetochore biorientation or chromosome congression, they do appear to inhibit the ability of a cell to perfectly biorient all its chromosomes.

### GSK-3 inhibitors alter spindle morphology

**Figure 6:**
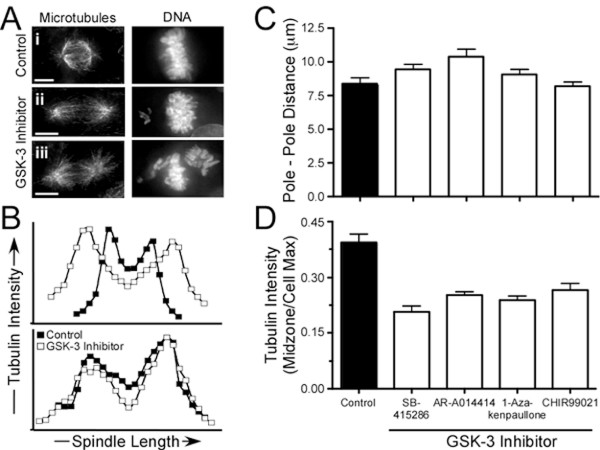
**GSK-3 inhibitors weaken the spindle midzone**. DLD-1 cells were incubated for 24 hours with GSK-3 inhibitors then fixed and stained to detect the microtubules and the chromosomes. (**A**) Projections of deconvolved image stacks showing representative mitotic spindles. Scale bar: 5 μm. (**B**) Graphs plotting tubulin intensity along the spindle axis. (**C**) Bar graphs plotting pole-pole distance. Values represent the mean and s.e.m. derived from at least 18 cells. (**D**) Bar graphs quantifying the tubulin intensity at the spindle midzone. Values represent the mean and s.e.m. derived from at least 15 cells. The cells in parts A-B were treated with either 2.5 μM 1-Azakenpaullone or 30 μM SB-415286

During the analysis described above (Figs 2B and 5), we noticed that in drug-treated cells, spindle morphology appeared abnormal in that there appeared to be increased astral microtubules near the pole around which chromosomes clustered. In addition, spindle length appeared to increase. Examples of both these phenotypes are shown in Figure 6A. In panel ***i***, the control cell shows a normal spindle morphology, with the chromosomes aligned between two robust crescent-shaped half spindles, and a small number of astral microtubules projecting away from the poles. In panel ***ii***, the spindle seems abnormally long, with the half spindles forming cones rather than crescents. In panel ***iii***, the pole on the right appears as a radial aster with equal numbers of microtubules pointing in all directions, rather than a crescent oriented towards the spindle equator.

To quantitate these differences, we measured tubulin fluorescence intensities from one end of the cell to the other along the spindle axis [48]. When plotted as a function of spindle position, the tubulin intensity typically gave two peaks, corresponding to the spindle poles, and a trough, corresponding to the spindle midzone (Fig. 6B). In some cells, the peaks were further apart in drug-treated cells indicating an extended spindle length (Fig. 6B, top panel). However, in other cases there appeared to be little difference (6B, bottom panel). When we measured the pole-to-pole distance in control cells, the mean value was 8.4 ± 0.4 μm. The mean values derived from GSK-3 inhibitor-treated cells were marginally higher, reaching 10.4 ± 0.6 μm in the presence of AR-A014418 (Fig. 6C). However that these differences are not statistically significant (Table 4), possibly reflecting a small effect or our small sample size.

**Table 4:** Effect of GSK-3 inhibitors on spindle morphology

**Parameter**	**Control**	**SB-415286**	**AR-A014418**	**1-Aza kenpaullone**	**CHIR99021**
Pole-Pole Distance (μm)	8.39 ± 0.45	9.45 ± 0.37	10.36 ± 0.57	9.06 ± 0.39	8.18 ± 0.33
	(22)*	(20)	(19)	(20)	(18)
	-	p > 0.05^§^	p < 0.05	p > 0.05	p > 0.05
Tubulin Intensity (midzone/cell max)	0.39 ± 0.02	0.21 ± 0.01	0.25 ± 0.01	0.24 ± 0.01	0.26 ± 0.02
	(15)*	(20)	(19)	(20)	(18)
	-	p < 0.001	p < 0.01	p < 0.001	p < 0.05
Interkinetochore Distance (μm)	0.91 ± 0.04	1.04 ± 0.03	1.05 ± 0.04	0.93 ± 0.02	0.92 ± 0.02
	(40, 4)	(33, 4)	(20, 3)	(52, 4)	(41, 4)
	-	p < 0.05	p > 0.05	p > 0.05	p > 0.05
Bub1 Intensity (Bub1:ACA)	100.00 ± 3.30	42.59 ± 0.85	24.24 ± 0.40	46.20 ± 0.98	68.50 ± 0.79
	(76, 4)^‡^	(64, 4)	(116, 3)	(81, 4)	(124, 5)
	-	p < 0.05	p < 0.001	p < 0.001	p > 0.05
BubR1 Intensity (BubR1:ACA)	100.00 ± 0.40	63.67 ± 0.30	31.49 ± 1.24	89.27 ± 0.75	84.08 ± 0.49
	(121, 7)^‡^	(115, 6)	(133, 4)	(176, 6)	(127, 5)
	-	p < 0.001	p < 0.001	p < 0.001	p < 0.001

Quantitation of spindle length (pole to pole distance), inter-kinetochore distance and kinetochore localisation of Bub1 and BubR1 in DLD-1 cells treated with GSK-3 inhibitors.

* Values in parentheses (Number of cells)

^‡ ^Values in parentheses (Number of kinetochores, Number of cells)

^§ ^Kruskal-Wallis Test (Nonparametric ANOVA), Dunn's Multiple Comparisons Test.

Tubulin intensity along the spindle length was, however, significantly different in drug-treated cells. In the examples shown in Figure 6B, the trough between the two peaks – which corresponds to the spindle midzone – is lower in both cases. Indeed, in control cells, the ratio of tubulin intensity at the midzone compared to the maximum value in that cells was 0.39 ± 0.02 (Fig. 6D, Table 4). By contrast, in drug-treated cells this value was reduced to between 0.21 ± 0.01 in the presence of SB-415286, and 0.26 ± 0.02 in the presence of CHIR99021. Thus, GSK-3 inhibitors do appear to affect spindle morphology.

### GSK-3 inhibitors reduce Bub1 levels at aligned chromosomes

The spindle checkpoint maintains chromosome stability by delaying anaphase until all the chromosomes are correctly bioriented [49-51]. That cells treated with GSK-3 inhibitors delay mitosis in the presence of unaligned chromosomes indicates that the spindle checkpoint is largely intact (Fig. 3). Consistently, when exposed to the spindle toxins taxol and nocodazole, SB-415286 treated cells arrest in mitosis (data not shown). However, the spindle checkpoint is capable of delaying anaphase in response to a single unaligned chromosome [51,52]. Because drug-treated cells often enter anaphase with unaligned chromosomes (Fig. 3), it is possible that the GSK-3 inhibitors suppress the checkpoint signal below the threshold required to detect a single unaligned chromosome. While many factors determine spindle checkpoint signalling [53,54], kinetochore localisation of Bub1 and BubR1 is required for full checkpoint function [37,55]. Therefore, we analysed the levels of kinetochore bound Bub1 and BubR1 following drug exposure.

**Figure 7:**
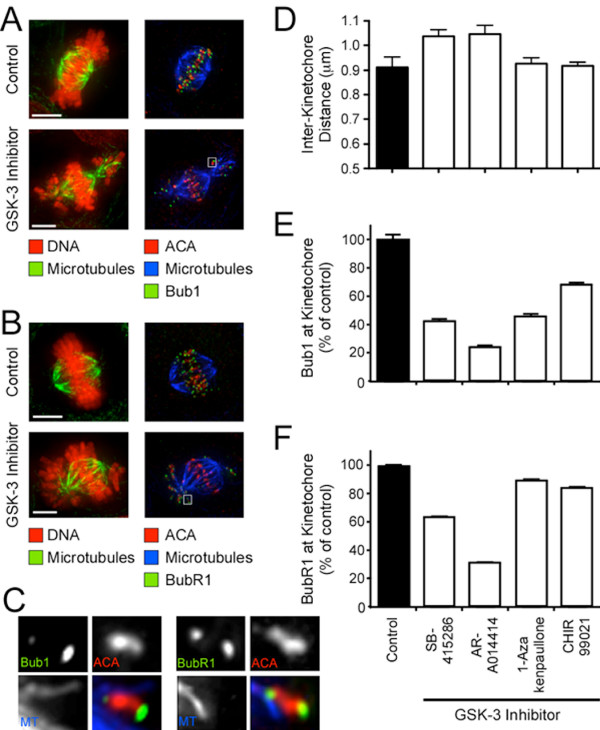
**GSK-3 inhibitors increase inter-kinetochore distance**. Drug-treated DLD-1 cells were fixed and stained to detect centromeres (ACA, red), kinetochores (Bub1 or BubR1, green), microtubules (green or blue) and the chromosomes (red) as indicated. (**A, B**) Projections of deconvolved image stacks showing reduced levels of Bub1 and BubR1 is at metaphase-kinetochores is reduced in GSK-3 inhibitor treated cells. Scale bars: 5 μm. (**C**) Projections of deconvolved image stacks showing the asymmetric labelling of Bub1 on the kinetochore of mono-orientated chromosomes. (**D**) Bar graph quantifying inter-kinetochore distance at metaphase chromosomes. Values represent the mean and s.e.m. derived from at least 20 kinetochores in at least 3 cells. (**E, F**) Bar graphs quantifying the levels of Bub1 and BubR1 at aligned chromosomes. Values represent the mean and s.e.m. derived from at least 64 kinetochores in at least 3 cells. The cells shown in (A) were treated with 30 μM SB-415286 and in (B) with 10 μM CHIR99021.

In control and drug-treated cells, Bub1 and BubR1 were recruited to kinetochores in prophase and prometaphase (not shown). In control cells, Bub1 and BubR1 persisted at metaphase kinetochores, albeit at reduced levels (Fig. 7A, B). In drug-treated cells, Bub1 and BubR1 were clearly present at the kinetochores of unaligned chromosomes. Consistent with our previous observations suggesting that Bub1 localisation is attachment-sensitive [36], Bub1 asymmetrically labelled the kinetochores of mono-oriented chromosomes, with the unattached kinetochore staining stronger (Fig. 7A, C). In addition, consistent with our previous notion that BubR1 localisation is tension-sensitive [36], BubR1 staining was more symmetrical at the kinetochores of mono-oriented chromosomes (Fig. 7B, C). Furthermore, pixel intensity-measurements revealed that the levels of Bub1 and BubR1 at these unaligned chromosomes were no different from unaligned kinetochores in control cells (data not shown). Thus, prior to chromosome alignment, the behaviour of Bub1 and BubR1 appear largely normal in the presence of the GSK-3 inhibitors.

The abundance of Bub1 and BubR1 at aligned chromosomes was however significantly reduced (Fig. 7A, B and Table 4). Pixel intensity-measurements showed that SB-415286, AR-A014418, 1-Azakenpaullone and CHIR99021 all reduced kinetochore bound Bub1, down to 25% in the case of AR-A014418 (Fig. 7E and Table 4). Kinetochore bound BubR1 was also reduced by all four drugs, most notably by SB-415286 and AR-A014418 (Fig. 7F and Table 4). One explanation for these observations is that the bioriented kinetochores had higher microtubule occupancy and/or were under more tension. To test this, we measured the inter-kinetochore distances of aligned chromosomes. In control cells, the mean inter-kinetochore distance was 0.91 ± 0.04 μm. In SB-415286- and AR-A014418-treated cells, this value increased to ~1.05 μm, a statistically significant difference (Fig 7D and Table 4). Although 1-Azakenpaullone and CHIR99021 had little effect, note that there is a correlation between inter-kinetochore distance and BubR1 levels. While SB-415286 and AR-A014418 increased inter-kinetochore distance and reduced BubR1, 1-Azakenpaullone and CHIR99021 had only modest effects on BubR1 binding and inter-kinetochore distance (Table 4). Significantly, SB-415286 and AR-A014418 also had more penetrant effects on chromosome alignment (Figs 2C, 3D) and pole-to-pole distance (Fig. 6C and Table 4).

### GSK-3β RNAi induces mitotic defects

**Figure 8:**
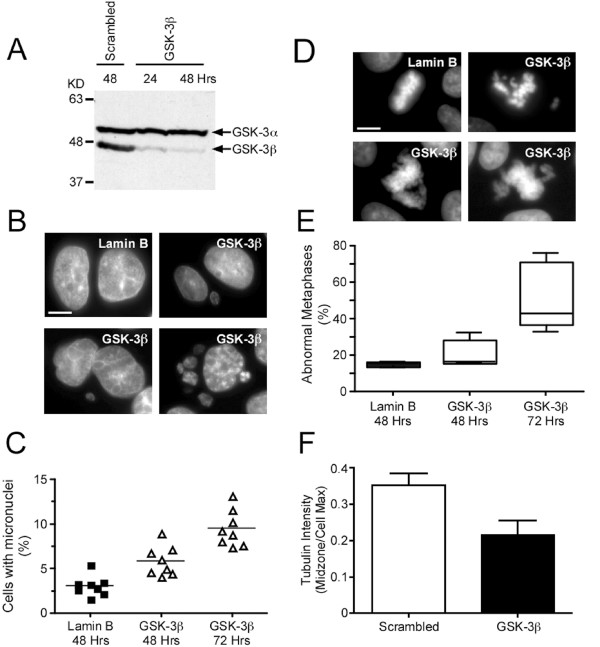
**GSK-3β RNAi inhibits chromosome alignment**. DLD-1 cells were transfected with siRNA duplexes designed to repress GSK-3β then analysed by immunoblot and immunofluorescence. (**A**) Immunoblot showing repression of GSK-3β. (**B**) Images of interphase cells showing micronuclei following repression of GSK3-β. (**C**) Scatter plot quantitating cells with micronuclei. The data is derived from four independent experiments, with each symbol representing an individual coverslip from which a minimum of 1,000 cells was counted. (**D**) Images of mitotic cells showing chromosome alignment defects in GSK-3β deficient cells. Bar = 5 μm. (**E**) Box plot quantitating abnormal metaphases in control (black bar) and GSK-3β RNAi (white bar) populations, 48 and 72 hours after transfection. The data is derived from three independent experiments. (F) Bar graphs quantifying the tubulin intensity at the spindle midzone. Values represent the mean and s.e.m. derived from at least 5 cells.

Our observations demonstrate that several different GSK-3 inhibitors effect spindle morphology and chromosome alignment, and elevate the chromosomes missegregation rate. Because we used a panel of structurally diverse compounds (Fig. 1), the simplest explanation is that the effects are due to inhibition of GSK-3. However, to rule out the possibility that the phenotypes observed may be due to off-target effects, we asked whether repression of GSK-3β by RNA interference yielded similar effects. We focussed on GSK-3β because this isoform localises to the mitotic spindle [33]. DLD-1 cells were transfected with siRNA duplexes designed to repress GSK-3β then immunoblotted with an antibody which recognises both GSK-3α and GSK-3β. Importantly, we observed a significant and selective reduction in GSK-3β levels (Fig. 8A). In the GSK-3β RNAi population, we frequently observed cells with micronuclei in the GSK-3β RNAi population (Fig. 8B), an indicator of chromosome missegregation [56]. Quantitation revealed that GSK-3β RNAi increased the number of cells with micronuclei by a factor of two 48-hours post transfection, and by almost 4-fold at 72 hours (Fig. 8C). Furthermore, we frequently observed metaphase spindles with multiple unaligned chromosomes (Fig. 8D). Quantitation showed a greater than 3-fold increase in such figures 72 hours after transfection (Fig. 8E). The spindle also appeared abnormal following GSK-3β RNAi, with an increase in astral microtubules (not shown) and an increase in spindle length; the mean pole-to-pole distance following repression of GSK-3β was 8.3 ± 0.4 μm compared to 7.7 ± 0.3 μm in control cells. Tubulin intensity at the spindle midzone was also significantly reduced (p < 0.05) following GSK-3β RNAi (Fig. 8F). BubR1 levels at metaphase kinetochores was significantly reduced; the mean BubR1/ACA ratio was 4.37 ± 0.94 in controls compared to 2.76 ± 0.24 in GSK-3β-deficient cells (p < 0.05). Inter-kinetochore distance was also significantly increased from 1.02 ± 0.03 μm in controls to 1.15 ± 0.04 μm following repression of GSK-3β (p < 0.05). Thus, as observed with the small molecule GSK-3 inhibitors, RNAi-mediated repression of GSK-3β affects spindle morphology, inhibits chromosome alignment and the fidelity of chromosome segregation.

## Discussion

### Small molecule GSK-3 inhibitors inhibit chromosome alignment

Here, we use small molecule inhibitors to probe GSK-3 function in cell cycle progression and mitotic chromosome segregation. Several inhibitors, including SB-415286, AR-A014418, 1-Azakenpaullone and CHIR99021, all significantly reduced phosphorylation of glycogen synthase in cells, indicating efficient cellular inhibition of GSK-3. Importantly, these four compounds inhibit chromosome alignment. Several other compounds, which reportedly inhibit GSK-3 *in vitro*, had little effect on glycogen synthase phosphorylation and had no effect on chromosome alignment. Why these latter inhibitors did not inhibit GSK-3 activity in cells is unclear but could be due to trivial reasons such as limited permeability. While the mode of action of these latter inhibitors is non-ATP competitive, the inhibitors which did inhibit GSK-3 activity in cells were all ATP competitors. This raises the possibility that their cellular effects may be due to inhibition of other protein kinases. However, all four inhibitors are relatively specific for GSK-3 inhibitors, at least *in vitro *[44,47,57,58]. Furthermore, they are all from different chemical classes and therefore likely to have different spectrums of "off-target" effects. Thus, the simplest explanation is that the chromosome alignment defect is due to inhibition of GSK-3. Indeed, the similarities between the GSK-3β RNAi phenotype and the inhibitor-induced phenotypes lends further weight to this notion. However, it will be important to test this hypothesis further in future studies, in particular the identification and characterisation of drug-resistant GSK3-β mutants will provide a powerful approach to dissecting the role of GSK-3 in cell cycle processes. In addition, analysing the effects these inhibitors have on GSK-3 null cells will help delineate off-target effects.

### Regulation of GSK-3 at the spindle pole

The observation that phospho-GSK-3α/β (Ser21/9) localises to spindle poles in mitosis [33], raises the possibility that GSK-3 is somehow involved in regulating microtubule dynamics and/or spindle pole function. Indeed, Wakefield *et al *showed that cells treated with two GSK-3 inhibitors, SB-2 and SB-4 affected spindle assembly and inhibited chromosome alignment. Here, we have extended the observations of Wakefield *et al *by (*a*) using a panel of diverse GSK-3 inhibitors, (*b*) demonstrating that mitotic phenotypes correlate with GSK-3 inhibition and (*c*) further characterising the mitotic defects. In addition, we show that cells treated with GSK-3 inhibitors enter anaphase with unaligned chromosomes. Consistent with Wakefield *et al*, our observations indicate that spindle morphology was frequently abnormal in cells treated with GSK inhibitors, with elevated numbers of astral microtubules. Interestingly, *in vitro*, GSK-3 phosphorylates another centrosomal kinase, Aurora A, on serines 290/291[59]. This in turn results in Aurora A auto-phosphorylation on serine 349. When S290/291 and S349 were mutated to phospho-mimicing aspartates, Aurora A was less active towards CPEB, a substrate that becomes phosphorylated in *Xenopus *oocytes following progesterone stimulation [59]. This phosphorylation event results in the translation of mos and Cyclin B mRNAs, which in turn drives cell cycle progression. Conversely, an Aurora A harbouring non-phosphorylatable alanines at 290/291 and 349 was constitutively active towards CPEB. Thus, GSK-3 appears to negatively regulate Aurora A, at least in *Xenpous *oocyte maturation. Whether GSK-3 phosphorylates Aurora A in somatic cells remains to be seen, but evidence from several systems indicate that Aurora A is required for spindle assembly [60-62], and recently, we have shown that Aurora A kinase activity is required for spindle assembly in human cells [63]. Because GSK-3 is inactive when phosphorylated on serine 21/9, it is possible that downregulation of GSK-3 at the spindle poles allows localised Aurora A activation, which in turn modulates microtubule function thereby facilitating spindle assembly. Whether GSK-3 is active away from the centrosome in mitosis remains to be seen, but it is conceivable that drug-mediated inhibition of GSK-3 could result in activation of Aurora A elsewhere in the cell, not just at the spindle pole. This in turn may stabilise microtubules resulting in the excessive asters observed in drug-treated cells.

### GSK-3, kinetochore – microtubule interactions and the spindle checkpoint

Cells treated with GSK-3 inhibitors align most of their chromosomes but have difficulty aligning them all and frequently enter anaphase with unaligned chromosomes. While the spindle defects may be sufficient to explain the chromosome misalignment, it is also possible that GSK-3 inhibition affects kinetochore behaviour. Support for this notion comes from the observation that the spindle checkpoint – which requires kinetochore function – is attenuated following GSK-3 inhibition. Clearly the spindle checkpoint is largely intact following GSK-3 inhibition: drug-treated cells delay anaphase onset in the presence of unaligned chromosomes (Fig. 4) and arrest when the spindle is destroyed with nocodazole (data not shown) However, drug-treated cells do enter anaphase with unaligned chromosomes, something which control cells never do. Furthermore, when HeLa cells were released from a G1/S block into SB-415286 plus nocodazole, the population mounted an attenuated checkpoint response, exhibiting a maximal mitotic index of ~40% (data not shown) compared to ~80% in controls (Fig. 3). Why the spindle checkpoint in GSK-3-inhibitor treated cells is attenuated remains to be seen: we did not observe any significant changes with respect to the levels of Bub1 and BubR1 at unaligned kinetochores (not shown). Interestingly however, levels of Bub1 and BubR1 were decreased at kinetochores of aligned chromosomes (Fig. 6). Because levels of kinetochore-bound Bub1 and BubR1 are indicators of microtubule occupancy and tension respectively [36], this suggests that GSK-3-inhibition actually promotes kinetochore – microtubule interactions. Consistently, inter-kinetochore distances increase in the presence of SB-415286 and AR-A014414. One possibility is that GSK-3-inhibition affects the spindle checkpoint via APC. Bub1 and BubR1 phosphorylate APC *in vitro*, an event which is enhanced if APC is already phosphorylated by GSK-3 [15]. Thus, following GSK-3 inhibition, phosphorylation of APC by Bub1 and BubR1 maybe attenuated.

Recently cells depleted of APC or expressing dominant negative mutants have been shown to have weakened collapsed spindles, defective kinetochore attachments, an increase in levels of kinetochore bound Bub1 and BubR1 and a decrease in tension across the kinetochore [16-18,21,64]. However, there appears to be no anaphase delay even though these cells go on and missegregate their chromosomes [65]. In contrast when GSK-3 is inhibited we observe an increase in spindle length and astral microtubules, a decrease in the level of kinetochore bound Bub1 and BubR1, an increase in the interkinetochore distance and a delay in chromosome alignment. This raises the possibility that GSK-3 negatively regulates APC's mitotic functions such that inhibiting GSK-3 activates APC, thereby yielding the opposite phenotype to APC depletion. Indeed inhibition of GSK-3 increases APC-microtubule interactions [13] which may help stabilise kinetochore-microtubule interactions.

## Conclusion

### GSK-3 and CIN

Our observations show that, at least in cultured cells, GSK-3 inhibitors induce chromosome non-disjunction, a phenotype that we also observed following RNAi-mediated repression of GSK-3β. Although GSK-3 inhibition affects spindle morphology, the chromosome alignment defect is the major contributing factor to non-disjunction. Is it possible that GSK-3 dysfunction causes chromosome instability in tumours? We feel that this is unlikely. Firstly, GSK-3 is rarely mutated in cancer [66]. Secondly, mouse knockouts are embryonic lethal [67]. Thus, in light of its essential and multi-faceted functions, GSK-3 dysfunction is likely to lead to cell death rather than provide a growth advantage. However, GSK-3 inhibitors are currently being investigated as potential therapeutics for a variety of chronic diseases such as diabetes and neurodegenerative disorders. Our observations suggest that GSK-3 inhibitors may have an unexpected consequence in these settings, namely the induction of chromosome instability, which may in turn promote tumourigenesis. Efforts to determine whether GSK-3 inhibitors induce chromosome instability in mouse models and patients seem to us to be imperative.

## Methods

### Cell culture and GSK-3 inhibitors

HeLa, HCT116, DLD-1 and DLD-1 cells stably expressing GFP-Histone H2B were all as described previously [38]. Cell culture conditions were as described [36]. Nocodazole (Sigma, 10 mg/ml in DMSO) was used at a final concentration of 0.2 μg/ml. The proteasome inhibitor MG132 (Calbiochem, 20 mM in DMSO) was used at a final concentration of 20 μM. RO-31-8220 (Calbiochem, 10 mM in DMSO) was used at a final concentration of 10 μM. SB-415286 (Tocris; 25 mM), TDZD-8 (Calbiochem; 20 mM), Inhibitor II (Calbiochem, 25 mM), OTDZT (Calbiochem; 31.8 mM), Inhibitor XI (Calbiochem; 25 mM), 1-Azakenpaullone (Calbiochem; 25 mM), AR-A014418 (Calbiochem, 25 mM), and CHIR99021 (kind gift from Philip Cohen; 10 mM) were dissolved in DMSO at the concentrations indicated, stored at -20°C in individual aliquots to avoid freeze thaw cycles, then freshly diluted in media. DMSO was added to control cultures to account for the solvent. Lithium Chloride (BDH) was dissolved in water at concentration of 1 M, filter sterilised and used at a final concentration of 40 mM.

### Antibody Techniques

Immunoblotting was done as described previously [21,36] using the following antibodies: rabbit anti-phospho-glycogen synthase (Cell Signalling Tech, 1:1,000); mouse anti-glycogen synthase kinase 3 (4G-1E, Upstate, 1:500); mouse anti-phospho-nucleolin (TG3, kindly provided by Peter Davies, 1:25); sheep anti-Bub3 (SB3.2, 1:1000). Immunofluorescence was done as described [21] using the following antibodies: TAT-1, mouse anti-tubulin (1:100, ref. [68]); SBR1.1, sheep anti-BubR1 (1:1,000, ref [36]); SB1.3, sheep anti-Bub1 (1:1,000, ref [36]); ACA, human anti-centromere (kindly provided by Bill Earnshaw, 1:800). To analyse kinetochore – microtubule interactions and spindle morphology, cells were permeabilised for 90 seconds in a microtubule stabilising buffer (100 mM PIPES pH 6.8, 1 Mm MgCl, 0.1 Mm CaCl_2_, 0.1% Triton X-100), fixed for 10 minutes in 4% formaldehyde, then blocked and incubated with the appropriate primary antibodies as described above. Following washes with PBST, cells were stained for 30 minutes at room temperature with the appropriate Cy2-, Cy3- or Cy5- conjugated secondary antibodies (Jackson Immuno-Research Laboratories), all diluted 1:500. Following washes, cells were stained with Hoechst 33358 at 1 μg/ml in PBST then mounted in 90% glycerol, 20 mM Tris-HCl pH 8.0. Deconvolution microscopy and pixel intensity quantitation was performed using a wide field optical sectioning microscope (Deltavision, Applied Precision) as described previously [36]. Briefly, for each cell, a Z-series of images at 0.2 μm intervals was captured at each wavelength and then processed using constrained iterative deconvolution. Deconvolved image stacks were projected and fluorescence signal intensities quantified using SoftWoRx (Applied Precision). To quantify the kinetochore bound protein, the average pixel intensities from at least 64 kinetochores from three or more cells was measured, background readings were subtracted and the values were then normalised against the ACA signal to account for any variations in staining or image acquisition. SoftWoRx was used to measure inter-kinetochore distance using Bub1 foci as indicated to determine kinetochore position. Tubulin intensity was determined as described previously [21,48]. Statistical analysis was performed using InStat^®^v3.0 (GraphPad Software Inc).

### Cell biology

DNA content and mitotic index measurements and synchronisation of TA-HeLa cells at G1/S using double thymidine block were done as described previously [37], but using an FSE-conjugated MPM-2 antibody (Upstate Biotech). At least 10,000 cells were then analysed on a FACSCAN (Becton Dickson). For time-lapse analysis, DLD-1 cells expressing the GFP-Histone fusion protein were cultured on either individual 35 mm glass bottomed Petri dishes or 35 mm glass bottomed 6-well plates (MatTek Co). Microscopy was performed on a manual Axiovert 200 (Zeiss), equipped with a PZ-2000 automated stage (Applied Scientific Instrumentation) and an environmental control chamber (Solent Scientific), which maintained the cells at 37°C in a humidified stream of 5% CO_2_, 95% air. Shutters, filter wheels and point visiting were driven by Metamorph software (Universal Imaging). Images were taken using a CoolSNAP HQ camera (Photometrics), while individual TIFF files were imported into Photoshop (Adobe) for printing or QuickTime (Apple) for movies. Nuclear envelope breakdown (NEB) was judged as the point when the prophase chromatin lost a smooth, linear periphery and the time of anaphase onset to be the first frame where coordinated pole wards movement was observed. Mitotic timing data is presented as box-and-whisker plots generated with Prism 4 (GraphPad), where the boxes show the median and interquartile range, while the whiskers show the entire range.

### RNAi

siRNA duplexes (SMARTpool, Dharmacon Research) designed to repress GSK-3β or non-specific control pool duplexes or siRNA duplexes designed to target lamin B1were transfected into DLD-1 cells using OligofectAMINE™ (Invitrogen) as describe [69]. In brief, 4 × 10^4 ^cells were seeded in 24-well plates 24 hours prior to transfection in growth media without antibiotics. siRNA duplexes were mixed with OligofectAMINE™ in OptiMEM media without antibiotics and incubated for 20 minutes. siRNA/lipid complexes were then added to cells for 6 hours followed by addition of complete media containing 20% foetal calf serum. 24 hours later the cells were replated onto coverslips or 6 well plates then analysed 24 hours later.

## Authors' contributions

Project conceived and experiments designed by AT and SST. Experiments carried out by AT assisted by AR and OS. Manuscript prepared by AT and SST.
